# Toxic confusion: the dilemma of antibiotic regulation in West German food production (1951–1990)

**DOI:** 10.1016/j.endeavour.2016.03.005

**Published:** 2016-06

**Authors:** Claas Kirchhelle

**Affiliations:** University of Oxford, Wellcome Unit for the History of Medicine, 45-47 Banbury Road, United Kingdom

## Abstract

• Cultural risk prioritization strongly influences antibiotic regulation.
• West German risk vernaculars and epistemes were residue-focussed.
• Hazards resulting from bacterial resistance selection were neglected.
• Successful bacterial resistance regulation depends on effective risk staging.

In November 2014, the German weekly *Die Zeit* confronted readers with a disturbing image. Staring at each other across the title page were a friendly looking pig and a human wearing a surgeon's mask. The image was titled “Revenge From the Sty.”^1^ Inside the issue, numerous articles warned about the overuse of antibiotics on German farms and the dire health effects of resistant pathogens. The November issue of *Zeit* marked the beginning of a series of *Zeit* reports dedicated to bacterial resistance and hygiene problems in food production and hospitals. According to *Correct!v*, the reporter collective behind many of the articles, the three most common multi-resistant pathogens (MRSA, ESBL, and VRE) were annually responsible for over 30,000 deaths and at least 1,000,000 infections in German hospitals. However, resistant bacteria were not limited to hospitals. Since the early 2000s, multi-resistant LA-MRSA CC398 had spread rapidly throughout German sties. In the intensive animal husbandry regions of Northern Germany, almost every third detected colonisation of humans with MRSA was defined as “livestock-associated” and almost 10% of infections detected in humans were caused by LA-MRSA CC398. Accordingly, the *Zeit* accused farmers and veterinarians of overusing antibiotics and endangering public health for the sake of cheap meat and quick profits.^2^

In response, furious farmers and veterinarians picketed the *Zeit* building in Hamburg. However, farmers failed to win public sympathy. In a 2014 *Zeit* interview, Minister of Agriculture Christian Schmidt went so far as to suggest restricting veterinary prescription rights. Six months later, the German government passed a new one-health based strategy to reduce bacterial resistance (DART 2020).^3^

How did it come to a situation where farmers were picketing newspapers and politicians were questioning veterinarians’ prescription rights? Now focussing primarily on bacterial resistance, German controversies about agricultural antibiotics are sixty-four years old. In 1951, antibiotics’ mass-introduction to West German agriculture enabled an unprecedented boom of animal production. It did not take long for side effects to emerge. Agricultural antibiotic use could cause residues in food and the environment, enable substandard welfare conditions, and select for bacterial resistance.

Faced with these hazards, Germans had to decide how to regulate the former miracle substances. However, different antibiotic hazards entail different regulatory responses: whereas it is sufficient to enforce drug withdrawal times to prevent residues in animal tissues and upgrade husbandry methods to improve welfare, only a permanent reduction of antibiotic use will curb bacterial resistance. Regulations’ shapes thus depend on which risks societies are most concerned about: residues in food, resistant pathogens, or maltreated animals.

Establishing antibiotic risk priorities is not straightforward. According to the sociologist Ulrich Beck, the modern category of risk has substituted absolute truth with hundreds of relative truths. Risk's virtual, probability based, anticipatory nature means that everything can be but nothing is risky. Risk is the anticipation of catastrophe but not the actual catastrophe. The anticipatory dimension of risk influences human expectations and triggers actions designed to avoid anticipated outcomes.^4^ Because of risk's half-known, anticipatory nature, objective measurements and authoritative expertise do not exist: “no one is an expert or everyone is an expert.”^5^ Ultimately, wider cultural evaluations decide whether a risk is seen as dangerous, urgent, real, or negligible. According to Beck, the “objectivity of a risk is the product of its perception and its staging. …. The risks we think we perceive and which scare us are the mirror image of ourselves and our cultural perceptions.”^6^

The concept of risk developed by Beck and others has given rise to a rich vein of literature on the commodification and distribution of risk and the changing and contested evaluation of risky substances and technologies.^7^ Some studies have stressed how distinct ways of producing knowledge have structured different ways of regulating risk. Focussing mainly on politics, legal systems, and experts, Sheila Jasanoff has highlighted how distinct “civic epistemologies” have produced different evaluations of controversial technologies in the US and Europe. Meanwhile, Alexander von Schwerin has pointed to the formation of risk epistemes amongst groups of scientists and resulting evaluations and representations of risk and risk policies. However, neither Jasanoff nor Schwerin extend their analysis to the wider cultural staging and evaluation of risks amongst consumers and lay users of risky technologies.^8^

This paper supplements research on civic epistemologies and risk epistemes with a focus on the development and impact of popular vernacular risk cultures.^9^ Building on the work of Heiko Stoff, Alexander von Schwerin, and Ulrike Thoms,^10^ this paper will show how the West German regulation of agricultural antibiotics was influenced by a widespread cultural *Angst* of adulteration and poisoning via invisible substances. By analysing reports in the national media, responses by the agricultural press, and decision-making by scientists and the federal government, it will become clear that West Germans’ *Angst* had deep-seated cultural-linguistic roots. Commonly referred to as *Chemie* (chemistry/chemicals) or—even worse—*Gift* (poison),^11^ many Germans associated agricultural antibiotics more with familiar toxic or carcinogenic chemicals and additives than with their use in medicine.

In turn, this toxicity- and carcinogen-focussed vernacular *Angst* influenced both scientific risk epistemes and regulatory decision-making. Whereas the public's *Chemie*-focus led to an early ban of antibiotic food preservation and an impressive residue-monitoring program, it also reduced political pressure for measures to combat bacterial resistance proliferation. Following a brief 1970s spurt of resistance-inspired regulation, West German attempts to address resistance proliferation remained stunted. Germany's focus on residues changed only gradually after reunification and led to a ban of antibiotic growth promoters (AGPs) by 2006. It is telling that in the context of the Transatlantic Trade and Investment Partnership, the German public seems to be more concerned about “toxic” US *Chlorhähnchen* (chlorinated chickens) than about permissive American antibiotic regulations.^12^

## It's a toxic world: West German antibiotic regulation 1945–1971

Although farmers were already using antimicrobial sulphonamides during the interwar period, it was not until the discovery of the so-called antibiotic growth effect that antibiotics’ mass-introduction to Western agriculture truly began. Publishing their findings in 1950, Thomas Jukes and E. L. Stockstad found that if consumed at low (subtherapeutic) doses, many antibiotics allowed animals to process feeds more efficiently and grow quicker. In addition to providing significant feedstuff savings, AGPs also promised to control bacterial herd infections. This latter effect was particularly important in the increasingly densely populated postwar sties.^13^

Whereas AGPs soon became extremely popular on US farms, West German antibiotic consumption initially lagged behind. After average daily caloric intake had fallen to 1100 calories during the “hunger-winter” of 1946–47, German officials attempted to boost food production whilst reintegrating thousands of refugees into the country's damaged fabric. As a consequence, many of the 11,571 new farms created in West Germany by 1950 were no bigger than ten hectares and not specialised on animal production. Although West Germany licensed antibiotics as “substances with special effect” for feeds in 1951, these were hardly conditions in which AGP use could flourish.^14^

However, major changes were afoot. Despite popular *Kulturkritik* of American mass culture,^15^ West German planners looked to the US for ways to intensify agriculture. During the early 1950s, enthusiastic agricultural media reports and exchange programs led many younger farmers to embrace US farming methods.^16^ Changes were particularly dramatic in the livestock sector where renewed access to international markets and cheap grain prices made many farmers concentrate entirely on animal production. By 1953–54, 70% of West German agricultural earnings resulted from selling animal products. Animal production was further fuelled by West Germany's economic take-off and concomitant *Fresswelle* (wave of gluttony). Whereas the average West German ate 37 kg of meat in 1950, this figure had doubled to 74 kg in 1968.^17^

In order to supply growing demand during times of rising labour costs and international competition, farmers began to invest in labour saving intensive animal production systems. Pioneered in the US, these year-round factory-like systems relied on regular antibiotic use to maintain herd health rather than veterinary treatment of individual animals. Unsurprisingly, both the US High Commission and American manufacturers propagated the adoption of US agricultural technology and sold production licenses for AGPs to West German companies.^18^

By the early 1960s, agricultural antibiotics had become a fixture in West German sties. In farming magazines, articles and commercials praised AGPs, antibiotic therapies, and numerous other applications.^19^ In 1966, the Minister of Agriculture estimated that about 80% of mixed feeds for young pigs, veal calves, and poultry contained antibiotic additives. AGPs remained banned for egg laying poultry, feeder cattle, and dairy cows.^20^ Although therapeutic doses were restricted to veterinary prescriptions, prescription-free AGPs could contain penicillin, spiramycin, oleandomycin, oxytetracycline, chlortetracycline, tetracycline, and bacitracin.^21^ By 1968, experts calculated that AGPs were annually saving German farmers 300–400 million marks in feed expenses.^22^ In 1970, one commentator exclaimed: “intensive mass-animal husbandry would be impossible without these mushroom-derived products.”^23^

Initially, commentators in the national press also expressed enthusiasm about antibiotics’ agricultural applications. Throughout the 1950s, the conservative *Frankfurter Allgemeine Zeitung* (FAZ), the left-leaning magazine *Der Spiegel*, and the liberal *Zeit* mostly praised medical and non-medical antibiotic-applications as part of a new “chemical age.” In 1954, the *Spiegel* enthused: “chemotherapeutics are now commonly available, and it is not much more difficult to kill a bacillus than to swat a fly.”^24^ Despite printing reports about antibiotic allergies and bacterial resistance in hospitals, early commentators failed to connect problems to antibiotic use on farms.^25^

However, by the second half of the 1950s, more sceptical comments began to appear. In 1956, the *FAZ* criticized new US antibiotic food preservatives: “The ‘marriage’ between medication and cutlet seems to us to be too unnatural to accept without opposition.”^26^ The newspaper's characterization of antibiotics as unnatural was significant. Although manufacturers like Pfizer stressed that antibiotics were derived from “natural” moulds,^27^ the German public categorised them as artificial *Chemie* (chemicals/chemistry). In the vernacular risk parlance of West Germans, agricultural antibiotics were therefore grouped in close linguistic association with other *Chemie* like hormones and food dyes. Over the following decades, agricultural antibiotics’ image would be tarnished via linguistic association with unrelated toxic or carcinogenic substances.

Fear of *Chemie* had deep roots in German culture. Since the nineteenth century *Lebensreform*, many Germans had participated in movements emphasizing the benefits of unadulterated food. During the interwar period, the growing focus on purer and therefore healthier lifestyles had given rise to numerous alternative agricultural and dietary schools. Common to many *Lebensreform* movements was the rejection of artificial *Chemie* such as fertilisers and pesticides in favor of “natural” circular farming. For supporters of alternative nutrition and agriculture, the adulteration of food and bodies was linked to degenerative diseases and cancer. German scientists contributed to these fears by increasingly distinguished between *Wirk*- and *Fremdstoffe* (active and foreign substances) in food. The Nazis, in their misguided quest for physical and racial superiority, also adopted elements of *Chemie*-free movements. Concerned about adulterated nutrition and carcinogens, the Nazis supported preventive health and dietary reform campaigns and research.^28^ However, as with other “green streaks” of fascism, the Nazi approach to preventive health and food purity was unsystematic and contradictory and wartime constraints often led to the abandonment of long-term health considerations.^29^

Nonetheless, interwar public and expert concerns about *Chemie* left a strong mark on Germans. With many supporters of food purity retaining influential posts, the postwar years saw a rapid resurgence of public *Chemie*-fears and an expert risk episteme firmly focused on the chemical adulteration of food and bodies. As early as 1949, a warning about the carcinogenic food dye butter yellow by Nobel laureate Adolf Butenandt triggered a large-scale letter campaign to President Theodor Heuß. During the early 1950s, fears of carcinogenic “Noxen” and a general condition of toxicity (*toxische Gesamtsituation*) fostered the establishment of numerous expert committees. Under the leadership of Adolf Butenandt and pharmacologist Hermann Druckrey, the German Research Foundation's (*Deutsche Forschungsgemeinschaft*, DFG) commission on food colorants (*Farbstoffkommission*) popularized Druckrey's so-called summation concept according to which even small doses of certain substances could cause cancer. The West German risk episteme was also successful internationally. West German experts soon regained leading roles within international bodies on cancer and food and Druckrey and his allies pressed for the establishment of an international list of “unobjectionable” food additives. In 1957, a European conference in Ascona—site of the famous vegetarian and nudist Monte Verità colony of artists, intellectuals, anarchists, and *Lebensreform* activists—developed an influential “positive list” of twenty-two unobjectionable food dyes.^30^

Druckrey's demands for a statutory list of approved substances found widespread public approval in West Germany and reinforced the popular risk vernacular surrounding *Chemie*. In 1956, the German Society for Nutrition called for a reduction of non-essential chemical food additives.^31^ The same year also saw a reprint of Curt Lenzner's 1931 influential *Gift in der Nahrung* (Poison in Food), which advocated a return to wholesome “pure” food.^32^ In 1957, the West German consumer council for nutritional questions bemoaned the “nonchalance with which the advances of the chemical industry are often seen abroad.”^33^

Resurgent *Chemieangst* also provoked attacks on antibiotic use in food production. The most active early group of West German critics was the *Gesellschaft für Vitalstofflehre* (society for vital substance teaching), which promoted scientific wholefood nutrition as a way to strengthen the body's natural defenses against to the hazardous synthetic artifice of modern civilization. Founded by the chemist Hans-Adalbert Schweigart, the *Gesellschaft* attracted many former Nazis but could later also point to Albert Schweitzer and Linus Pauling as prominent supporters. In 1955, it called for more research on agricultural antibiotics. Members of the *Gesellschaft* were concerned about bacterial resistance but mostly worried that antibiotics would disrupt the intestinal flora's vital powers.^34^ Werner Kollath was amongst the most prominent antibiotic critics. An inventor of the wholefood diet and former Nazi, Kollath's 1942 book *Die Ordnung unserer Nahrung* warned against the degradation of living food through processing. Postwar, Kollath was thus naturally critical of antibiotics’ growing use in food production. In 1959, he wrote a letter to the *FAZ* accusing the Munich veterinarian and animal nutritionist Johannes Brüggeman of trivializing AGPs’ hazards. Kollath warned that antibiotics selected for temporary resistance, destroyed the natural intestinal flora, and reduced meat's nutritional quality.^35^ However, Kollath's *Vitalstoff*-focused warnings proved ineffective.

Instead of addressing concerns about resistance or vital substances, the passing of the 1958 West German food law (*Lebensmittelgesetz*) once again revealed the power of antibiotics’ cultural association with *Chemie* and both reflected and reinforced the vernacular risk focus on *Chemie.* Despite many similarities to contemporary US legislation,^36^ one of the German food law's notable bans concerned antibiotic preservatives. Ahead of the 1958 reform, Prof. S. Walter Souci, head of the West German Research Institute for Food Chemistry, gave a long interview to the *Spiegel* in which he referred to a widespread German *Chemikalienfurcht* [*Chemie*-fear]. Significantly, Souci did not disagree with Germans’ *Chemikalienfurcht*. The new food law would permit substances only after manufacturers had shown them to be safe. Adopting tropes of contemporary *Kulturkritik*, Souci rejected the Anglo-American focus on consumer choice and recommended “re-educat[ing]” consumers to appreciate “natural”^37^ food and its colors.

Amongst the substances Souci specifically opposed were antibiotic food preservatives. Already licensed in the US and Canada and under consideration in the UK, tetracycline ice and dips were used to preserve fish, shellfish, and poultry. However, Souci noted that German regulators had “fundamental concerns” about adding such “extraneous substances” to “essential foodstuffs [*Grundlebensmittel*].”^38^ Ahead of his *Spiegel* interview, Souci had been part of a West German delegation to the US. Following its return, the delegation warned: “It is absolutely necessary to maintain the ban of antibiotics and hormones and more clearly differentiate the [law on animal feeds from the food law].”^39^ When discussing liberal US chemical use, a delegation member pointed out that, in contrast to Germany, US lawmakers “work[ed] on the premise of the correctness of all studies conducted by industry.”^40^ Although the German delegation was aware of US consumer concerns, it noted that these “did not have the same importance in the opinion of the wider public as in Germany.”^41^

Passed in December 1958, section 4b of the West German food law banned the use of antibiotic preservatives. Officials justified their decision by referring to antibiotics’ selection for resistant pathogens on food and residues’ allergenic effects. Other substances banned by section 4b were potentially carcinogenic preservatives, pesticide residues, and hormones with thyreostatic effects.^42^ The German association of housewives (*Berufsorganisation der Hausfrauen*) greeted the new law: “For the housewife, foreign substances are everything that is not contained in food from the start, i.e. also vitamin and provitamin additives, chemical smell and taste additives,.. and of course hormones and antibiotics….”^43^

Despite referring to the selection for bacterial resistance to justify banning antibiotic preservation, officials did not express concern about similar resistance selection on farms. Instead, the issue of bacterial resistance remained linked and limited to antibiotics’ immediate presence in dips, ice, the gut, or food. When asked about AGPs in 1958, Souci deemed the emergence of harmful resistant bacteria “rather unlikely” and was far more concerned about “a potentially toxic effect”^44^ of antibiotic residues and metabolites in foodstuffs. This emphasis on antibiotics’ unknown metabolites and view of bacterial resistance reflected the contemporary state of knowledge. Aware of antibiotic resistance since the early 1940s, researchers thought that bacterial resistance proliferated only vertically in a hereditary fashion. Bacteria were either naturally resistant or developed resistance through random mutations. Whereas antibiotics selected for resistant bacteria, discontinuing antibiotic use would end this evolutionary advantage and lead to resistant bacteria's competitive inhibition by sensitive bacteria. The vertical or “genetic” interpretation of bacterial resistance was further strengthened by the contemporary Lysenko affair's discrediting of “adaptationist” explanations of resistance. In contrast to later environmental concepts of “horizontal” resistance proliferation, thinking of resistance proliferation as the spread of individual bacterial strains made it easy to conceive of resistance as a phenomenon that was limited to certain locales. Because resistant strains would be subject to competitive inhibition once outside of antibiotic environments, 1950s experts could easily express concern about spreading resistance in hospitals whilst remaining unconcerned about similar resistance selection on farms. Meanwhile, the rapid development of new antibiotics made observers confident about humans’ ability to stay ahead of bacterial resistance.^45^

The combination of regulators’ and experts’ residue-focused risk episteme, the public's *Chemie*-oriented risk vernacular, and the theory of vertical resistance proliferation helps to explain why AGPs were licensed with such ease throughout the 1950s. As described by Ulrike Thoms, the majority of West German experts remained enthusiastic about antibiotics’ ability to boost national and global food production and help farmers survive growing competition.^46^ Attending influential symposia in the US, German researchers estimated that AGPs produced a surplus of 17.50–27.50 Marks per 100 kg of meat.^47^ Further aiding AGPs’ rapid licensing was the confused state of West German drug and feed legislation. Until 1961, West Germany had no unified drug law and the new law was quickly made obsolete by the contemporaneous thalidomide scandal. It was only in 1976 that a new drug law created a mandatory licensing system based on efficacy and safety controls.^48^ Still based on rules from 1926, similar confusion characterized German feed regulations. After the 1951 *Futtermittelnormordnung* (feedstuff norm regulation) licensed antibiotics in feeds as “substances with a special effect,” AGP manufacturers could send permit petitions to a Ministry of Agriculture expert committee. However, permit evaluations cannot have been too demanding since the number of special permits, permit modifications, and other rulings had grown to 17,121 by 1967.^49^

Meanwhile, officials had virtually no oversight of antibiotics’ use on farms or presence in foodstuffs. In 1968, Prof. Dieter Großklaus of the Federal Health Office [*Bundesgesundheitsamt*] warned about “absolutely insufficient” assay methods and called for an “encompassing residue control for German animal products.”^50^ However, Großklaus’ warnings went unheeded. Instead, officials and media commentators boasted about the superiority of West German food safety and complained about lax foreign standards.^51^ This would not stay so for long.

In Britain, unique research facilities were gradually shifting concerns away from residues to the issue of bacterial resistance proliferation. Starting in the mid-1950s, British scientists published alarming data on the spread of resistant bacteria on farms and in hospitals.^52^ By the mid-1960s, concerns were heightened by findings that resistance not only proliferated *vertically* but could also be communicated *horizontally* between different bacterial species via the exchange of extrachromosomal DNA fragments called plasmids. Plasmid-borne resistance (R-factors) spread in an “infectious” manner and turned bacterial resistance from a local into an environmental hazard.^53^ Alarmed, the British government convened two so-called Netherthorpe committees on agricultural antibiotics, which called for a change of licensing in favor of nontherapeutic (therapeutically irrelevant) antibiotics in 1962 and a review of veterinary prescription practices in 1966.^54^

German reactions to the Netherthorpe reports were mixed and mostly linked the dangers of resistance to the immediate presence of antibiotic residues. During the early 1960s, influential researchers like Hans Knothe from the University of Kiel claimed that there was no evidence of harm resulting from AGP-use. Knothe's position was endorsed by the German Congress of Physicians (*Ärztetag*) in 1959 and by the Federal Health Council (*Bundesgesundheitsrat*) in July 1961. In 1963, Knothe claimed that in the case of tetracycline, resistance development in humans became relevant only when exposure exceeded 25 mg per day. A decade of experience had shown that allergies would not emerge because AGPs were too low-dosed and residues were destroyed by cooking.^55^ Media reports on antibiotic resistance also remained marginal. Whereas one *FAZ* article warned against resistance development,^56^ another portrayed antibiotics as natural substances also “contained in natural feed- and vegetable plants, for example nasturtium.”^57^ The latter article criticized Germans’ adherence to the false binary of “’natural’ and ‘good”’ vs. “’unnatural’ and ‘bad’.”^58^ In 1964, Ruth Harrison's *Animal Machines* [translation 1965]^59^ triggered West German protest against animal welfare problems and antibiotic overuse on “factory farms.” However, once again, the main issue was not the selection for bacterial resistance but antibiotics’ effect on food quality and safety. In 1966, a *Spiegel* article's almost literal translation of *Animal Machines* described agricultural antibiotics as a ticking “biological bomb”^60^ but continued to limit the hazards of resistance proliferation to antibiotic residues’ immediate presence. The *Chemie*-focus proved hard to break.

It was only in 1967—over one year after reports in the Anglo-American press—that the *Spiegel* warned its readers about plasmid-borne R-factors as “tentacles of horror.”^61^ Citing the *New England Journal of Medicine*, the *Spiegel* accused the “tons (*Tonnen-Quantitäten*)”^62^ of agricultural antibiotics of facilitating a return to pre-antibiotic times. In 1968, the *Zeit* followed suit and warned about resistant epidemics resulting from overuse of agricultural antibiotics. Describing plasmids as a kind of “bacterial esparanto,”^63^ the newspaper enumerated policy measures to counteract resistance such as mandatory antibiotic withdrawal periods prior to slaughter and a reservation of therapeutically relevant antibiotics for humans. However, despite its belated acknowledgment of “dangerous” R-factors, the West German press continued to frame antibiotic hazards mainly as invisible residues.^64^ “Infectious resistance” emanating from farms thus failed to change the traditional risk vernacular's focus on chemical residues. The staging of the new risk had been unsuccessful.

West German farmers shared the national media's culturally ingrained focus on *Chemie*-residues. Although most farmers reacted hostile to consumer criticism, farming magazines featured many articles warning about exposure to *Chemie* in food and the environment. On fields and in sties, *Chemie* was considered beneficial, in human bodies *Chemie* was equated with *Gift*.^65^ This rule also held true for agricultural antibiotics. Lacking national residue controls, dairies and magazines informed farmers how to avoid antibiotic residues in milk when treating cows for mastitis (a wide group of bacterial udder infections). By contrast, warnings about bacterial resistance rarely featured.^66^

However, major changes were afoot. In 1969, Britain announced pioneering resistance-motivated AGP bans. Compelled by scientific and public pressure, the British government had convened the so-called Swann Committee in 1968. One year later, the Swann Committee's report called for a ban of penicillin and tetracycline AGPs. Only antibiotics considered medically irrelevant were to be included in AGPs. Although resulting reforms failed to reduce either antibiotic use or bacterial resistance, the British Swann report heralded a major shift of antibiotic regulation and put significant pressure on other European governments to reform their antibiotic regulations.^67^

In Germany, reform calls were endorsed by the DFG and led to the formation of a new expert and regulatory risk episteme focusing on the hazards of bacterial resistance. In 1967, the DFG Commission on Active Ingredients in Animal Nutrition (*Kommission für Wirkstoffe in der Tierernährung*) under veterinary pharmacologist Helmut Kewitz had begun to canvass experts’ opinions on AGPs.^68^ Published only slightly after Swann, a 1969 report by the Kewitz Commission warned that none of Germany's AGPs fulfilled pre-defined DFG criteria. Feed antibiotics should:

1) be resorbed only in small quantities in the gastro-intestinal tract;
2) not effect changes in animals’ bodies, which would complicate residue detection;
3) not possess antigenic or haptic properties [*Hapteigenschaft*], so that allergies would not occur;
4) not be used as therapeutics for humans and animals and not select for cross- and multiresistance to therapeutic antibiotics.^69^

Criticizing penicillin's allergenic properties, the Commission reported that DFG-commissioned microbiological evaluations had confirmed the danger of “infectious” (multi)-resistance emanating from AGP-fed animals. It was necessary to “assume that [AGPs] would reduce the therapeutic efficacy of antibiotics”^70^ for human and animal diseases—especially those associated with *enterobacteriacae*. The Kewitz Commission recommended “no longer delaying unavoidable decisions.”^71^ West Germany should restrict nonmedical antibiotic use and only license nontherapeutic AGPs that did not select for multiple resistance amongst intestinal bacteria. With the exception of penicillin feeds, which were to be banned immediately, the Commission recommended a transitional period of three to four years for the introduction of AGP-restrictions. It was hoped that this would foster the development of nontherapeutic AGPs. Other recommendations included reducing antibiotic use during animals’ finishing period, mandating DFG-approved antibiotic dosages, and avoiding further expansions of antibiotic use.

## The age of the *Chemie-Bauer* (1970–1989)

Although it endorsed the Kewitz report and banned penicillin AGPs as early as 1969, the West German government could not enact further restrictions on its own. As a member of the European Economic Community's (EEC) Common Agricultural Policy (CAP), West Germany had agreed to harmonize its veterinary drug, feed, and food laws with the rest of the community.^72^ Launched in 1967 and supposed to come into effect in 1969, initial EEC harmonization policies were supposed to unify labels, drug dosages in feeds, and tolerance levels for residues in animal tissues. While initial EEC harmonization had not addressed antibiotic resistance or restrictions, the Swann and Kewitz reports prompted the EEC to pass Directive 70/526/EEC in November 1970. The directive gave member states up to six years to phase out penicillin, tetracyclines, and the macrolide spiramycin from AGPs.^73^ In West Germany, tetracycline AGPs were phased out by August 1973 and streptomycin AGPs’ license expired in 1974. Reasserting the trope of superior German standards, a federal official proclaimed that West Germany was “probably the state with the least antibiotic classes used in feeds.”^74^

**Fig. 1:**
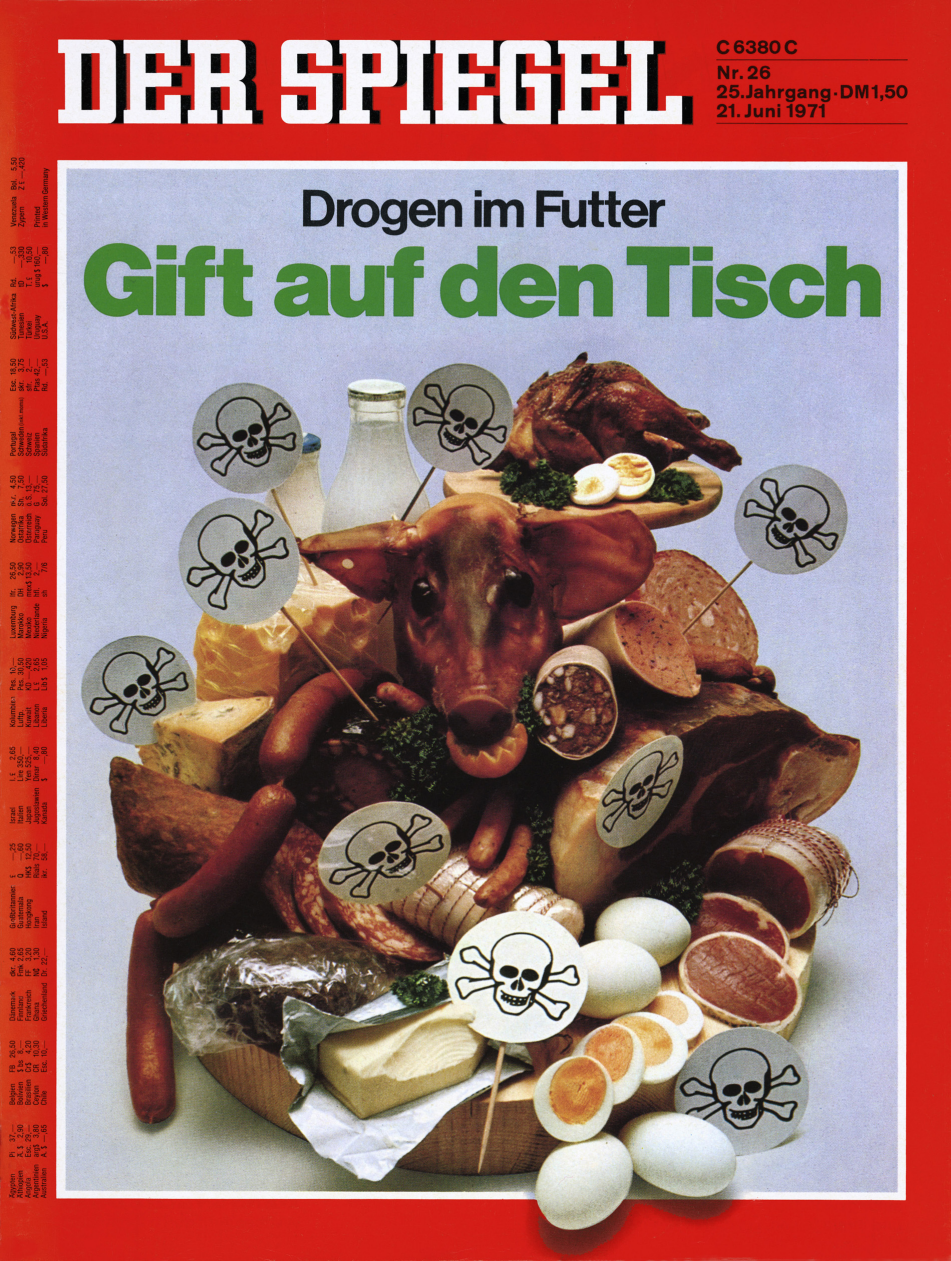
Cover of *Der Spiegel* 26 (1971) “Gift auf dem Tisch.” http://www.spiegel.de/spiegel/.

However, German media commentators did not think that phasing out AGPs went far enough. Instead, the 1970s and 1980s saw an intensification of *Chemie*-fears and portrayals of farmers as irresponsible *Chemie*-*Bauern* (chemical farmers). Regarding antibiotics, the resurgent popular risk vernacular of *Chemie* residues once again overshadowed the risk of resistance proliferation and shaped German antibiotic regulation. In 1971, a *Spiegel* cover featured a table laden with meat products bearing poison (*Gift*) signs (Fig. 1). The issue was titled “Drugs in the feed, poison on the table.”^75^ Despite the coming AGP restrictions, the *Spiegel* claimed: “the drug [*Die Droge*] is still omnipresent in [animals’] trough.”^76^ With legal and illegal access to many dangerous substances, farmers were endangering consumers. Farmers’ use of the toxic antibiotic chloramphenicol “was as dangerous as the razor in the hand of the monkey.”^77^ Referring to “black market channels” and invoking the specter of cancer, an “industry expert” warned: “nobody controls to what extent crunchy cutlets and roast chickens from the grill contain dangerous residues.”^78^ With slaughtered animals supposedly “full of antibiotics to their neck,”^79^ the magazine clamed that a recent study had found antibiotic residues in over 67.3% of 1508 veal carcasses. Despite noting that up to 10% of human coli-bacteria were already resistant to more than five antibiotics, the *Spiegel's* main concern was clearly that Germans were “unprotected from residues of penicillin in their breakfast milk, from carcinogenic arsenicals in their crunchy cutlet.”^80^ In the *Zeit*, Theo Löbsack reported that illegal veterinary drug sales now accounted for 40–50 million Marks or 30–40% of the total market.^81^ The *FAZ* printed similar reports.^82^

The intensification of German concerns about antibiotic residues was no coincidence. In 1972, newspapers had reported a major residue scandal in Bavaria. Between 1970 and 1972, the city of Munich had analyzed 19,000 animal carcasses for antibiotic residues. According to the Director of Communal Services, Werner Veigel, the results were “simply scandalous”: signs of veterinary drug abuse and residues had been detected in 12% of cattle, 22% of calves, 7% of pigs, and 3% of sheep.^83^ Downplaying Veigel's findings and putting pressure on the defiant official, the Bavarian government wrongly claimed that tests had included so-called casualty slaughters and that ordinary Bavarian meat was unobjectionable.^84^

Prompted by Veigel's warnings, an EEC information request disclosed that Bavarian histological analyses had shown that 27% of male and 56% of female calf carcasses indicated exposure to illegal carcinogenic estrogens.^85^ Meanwhile, the Bavarian Veterinary State Research Institute found antibiotic contamination in 23.9% of fresh meat, 32.6% of meat products, and 3.2% of milk samples.^86^ Internally, West German officials acknowledged that there was “no doubt that animals and fresh meat contained a certain extent of antimicrobial substances.”^87^ However, the supposedly strict West German meat inspection (*Fleischbeschaubesetz*) and food laws (*Lebensmittelgesetz*) contained no clause allowing officials to penalize offenders. Section 4b Nr. 1 of the 1958 food law prohibited antibiotics’ deliberate addition to food but did not address their “accidental” presence in meat. In contrast to the milk law (*Milchgesetz*), which mandated a five-day antibiotic withdrawal period and outlawed residues in milk, officials had to prove to courts that individual residue amounts found in meat would endanger consumers’ health.^88^

With food security compromised and residues also found in meat imports, Bonn was once again forced to act. In 1972, social democratic Minister of Health Käte Strobel, who had been one of the key forces behind the 1958 food law, promised to upgrade meat controls, reduce antibiotic concentrations in feeds, and introduce mandatory drug withdrawal times.^89^ Reflecting the public's on-going risk prioritization of residues, officials, however, did not choose to monitor for bacterial resistance. They also considered further antibiotic restrictions “unacceptable” because they expected “the strongest resistance [from farmers].”^90^ Concerned about agricultural protest, a memo advised the Bavarian government “to avoid everything that could distract the public from the sole responsibility of the federal government for the solution of the problem.”^91^

Following bitter internal clashes over antibiotics’ respective hazards, the West German government launched a national residue-sampling program using bacterial inhibition tests in April 1974.^92^ Inhibition tests are quick and easy to use and follow a simple principle: kidney and muscle tissue samples are placed in a petri dish containing culture medium and a test organism (usually *Bacillus subtilis*). If, following a pre-defined incubation period, bacterial growth around meat samples is inhibited this can indicate antimicrobial residues, which can then be tested for with more precise but slower methods.^93^ In West Germany's public abattoirs, inhibition tests were to be used on every 100th animal carcass and every casualty slaughter. Between April and December 1974, veterinary officials conducted a staggering 165,000 inhibition tests resulting in 1.5% positives for muscle meat and 10% positives for offal samples. The 132-fold of contemporary federal testing in the US, the massive scale of testing was supposed to restore trust in West German meat.^94^

In the agricultural community, inhibition tests remained controversial. For one thing, regulators initially had difficulties assessing how big inhibition zones had to be to indicate drug residues as opposed to naturally occurring antimicrobial substances. Comparing the inhibition test to a “lottery game,”^95^ this imprecision caused alarm amongst farmers fearing false positives. Moreover, early *B. subtilis*-based tests’ sensitivity varied significantly for different antibiotics:

**Table:** 

Antibiotic	Inhibitory concentration
Chlortetracycline	0.05 ppm
Tetracyclin	0.3 ppm
Oxytetracycline	0.6 ppm
Penicillin	0.1 ppm
Tylosin	10 ppm
Erythromycin	6 ppm
Spiramycin	150 ppm
Oleandomycin	10 ppm
Streptomycin	8 ppm
Neomycin	100 ppm
Chloramphenicol	5 ppm
Polymixin	300 ppm^96^

The public health implications were significant. Tests were able to detect small concentrations of legally available and nontoxic tetracyclines but only very high concentrations of hazardous black market antibiotics like chloramphenicol, neomycin, and polymixin. Noting that the odds of being caught remained small, one farming magazine claimed: “Shrewd or irresponsible people will continue to be able to get away with using the ‘right’ substances.”^97^ In other words, inhibition tests might actually boost black market sales.

Under public pressure to combat illegal *Chemie* in food, the West German government pressed for EEC-wide inhibition test controls. By the second half of the 1970s, West German pressure led to major clashes with the British government and German rejections of contaminated British meat.^98^ Describing a 1978 meeting on standardized EEC meat controls, a British delegate noted that discussions of sample sizes led to a “good deal of acrimonious discussion with the German representative proving the most vocal.”^99^ Continental representatives had talked “a good deal of nonsense … about the willingness of consumers to pay for extra protection.”^100^

Domestically, German regulators also attempted to crack down on the “Grey Drug Market” (*Grauer Arzneimittelmarkt*). Attempting to cut costs, some farmers were mass-medicating their herds with dubious drug cocktails. Meanwhile, various salesmen and veterinarians offered prescriptions and medications at competitive prices without even seeing the respective animals. By 1975, the popular term *Autobahntierarzt* (motorway veterinarian) had emerged to describe veterinarians selling drugs out of cars at motorway stops. The Grey Drug Market itself was enabled by a lack of nationally unified regulations for veterinary drug sales and a 1964 Dutch law legalizing the export of therapeutics even if these substances were illegal in other countries.^101^

Attempting to redress the situation without severing farmers’ access to prophylactic treatments, the West German government devised a compromise solution. In April 1974, a veterinary amendment (*Tierarztnovelle*) mandated that drugs should be dispensed to farmers only if herds had actually been inspected. One year later, section 12 of the 1975 ordinance for the purchase, production, storage, and distribution of therapeutics (*Verordnung über Erwerb, Herstellung, Aufbewahrung und Abgabe von Arzneimitteln*) specified that larger quantities of medication were to be dispensed only if animals were inspected every eight weeks and medications’ purpose and duration was documented prior to sales.^102^

However, the directives proved ineffective and reports soon surfaced that veterinarians were now selling drugs alongside “herd inspection confirmations” via mail order. A spot check in a Bavarian county revealed that “in no single case … use and fate of sold therapeutics”^103^ had been documented properly. Such erroneous documentation was not only problematic for food security but also regarding the state's ability to tax veterinarians’ income from drug sales. Amongst farmers and veterinarians, officials encountered a wall of silence:

It is usually impossible to follow up on occasional accusations (…) regarding the supposedly flourishing therapeutic black market because no single veterinarian is willing to file an official complaint.^104^

The silence was normally broken only when one veterinarian attempted to expand sales amongst another veterinarian's clients. However, even then, denunciations were filed privately. According to one veterinarian, farmers were purchasing medications directly because they “did not want to pay the 25 Marks for a herd visit from the responsible veterinarian.”^105^ By 1981, German states were conducting 400 investigations against illegal veterinary drug sales with most investigations centring on Lower Saxony, Bavaria, and Hessia. Most illegal sales consisted of beta-blockers, estrogens, sulphonamides, and antibiotics with chloramphenicol receiving special mention.^106^

Compliance problems also affected the West German feed industry. Despite increased fines and the incorporation of consumer protection into the federal feed law in 1973, local authorities often treated feedstuff offences as non-prosecutable minor misdemeanors. One reason for this was lacking access to antibiotic assays and widely differing assay methodology across West Germany.^107^ In 1973, spot checks revealed that 45.8% of antibiotic feed samples failed to meet German feed law requirements.^108^ While federal violation detections fell to 9.1% of analyzed samples in 1978, actual fines for feedstuff offences remained puny.^109^

Preoccupied with antibiotic residues and feed violations, German authorities continued to neglect the dangers of bacterial resistance proliferation and unilaterally licensed further AGPs such as avoparcin in 1977. Focusing on avoparcin's efficacy, toxicity, and carcinogenic effects, regulators ignored grave internal concerns about Cyanamid-sponsored resistance tests, which had measured resistance development amongst already resistant bacteria.^110^

Meanwhile, German consumers’ on-going concern about conventional food invigorated the previously marginal organic sector. For many consumers, a large part of organic food's appeal was the absence of *Chemie*. Regarding animal products, the absence of antibiotics and hormones served as a common denominator of many alternative production philosophies. Just how powerful the appeal of antibiotic-free “purity” could be is highlighted by the Redlefsen KG's 1972 launch of the antibiotic-free *Reinschmecker* (pure taster) sausage—a word play on *Feinschmecker* (gourmet). Described by Ulrike Thoms, the Redlefsen KG was subsequently sued and forced to end *Reinschmecker* sales by competitors fearing for the image of their own products.^111^ However, the *Reinschmecke*r-case did not end the organic boom and press articles continued to praise non-intensively farmed meat and “poison and chemical free agricultural products.”^112^ In 1981, the Catalyst-Group published the popular *Chemie in Lebensmitteln* (chemicals in food), which provided consumers with information on foodstuffs’ ingredients and how to avoid *Chemie*-exposure. Antibiotics featured prominently in the book.^113^

**Fig. 2:**
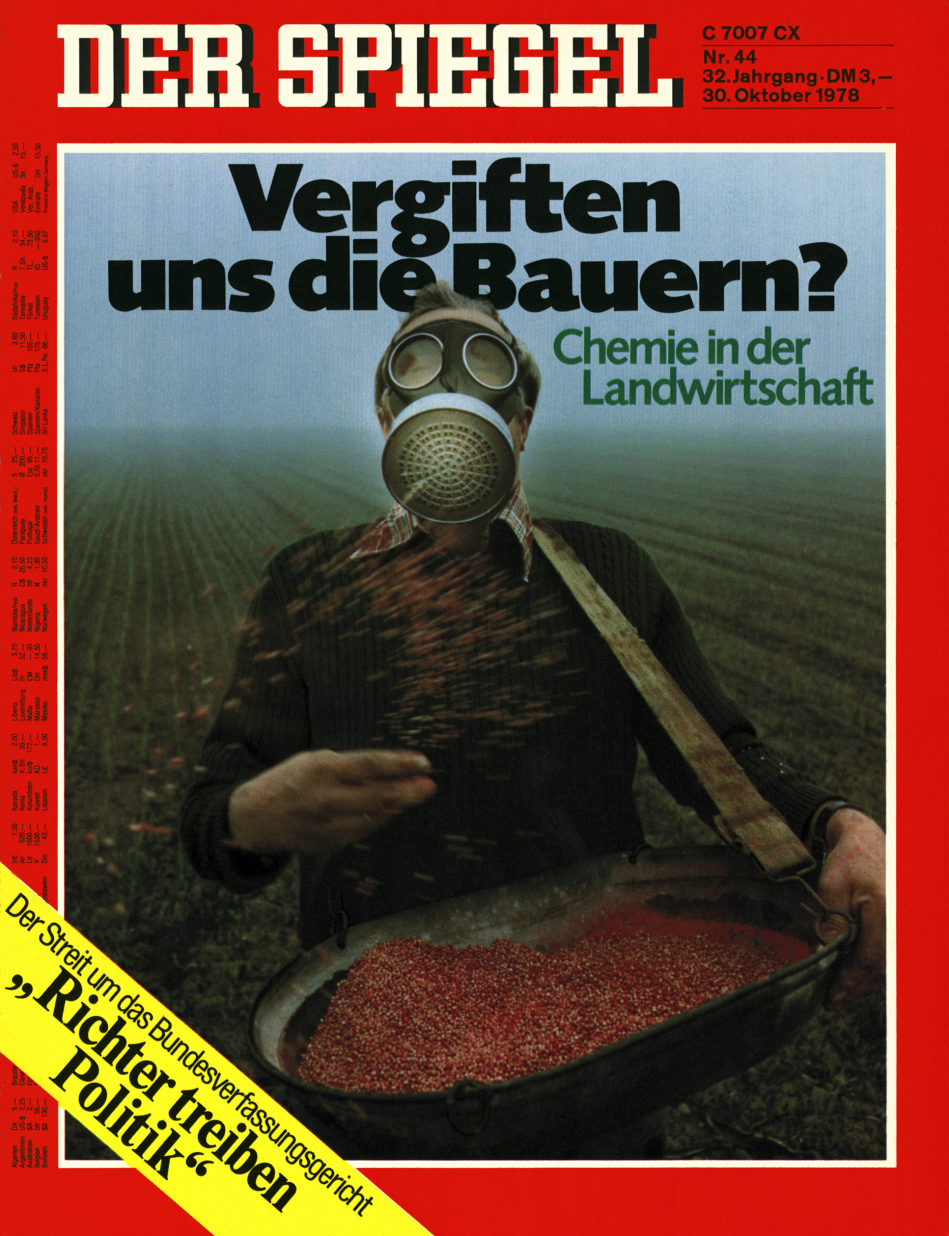
Cover of *Der Spiegel* 44 (1978) “Vergiften uns die Bauern?” http://www.spiegel.de/spiegel/.

With sales of “pure” food booming and *Chemie*-fears peaking in the years following the 1976 dioxin disaster in Seveso,^114^ West Germans continued to marginalise the hazard of bacterial resistance selection on farms. Titled “Are the farmers poisoning us? *Chemie* in agriculture,”^115^ a 1978 *Spiegel* issue featured a gasmask-wearing farmer sowing a field (Fig. 2). According to the *Spiegel*, mass-use of pesticides, antibiotics, and herbicides by *Chemie*-farmers was laying the table for Germans’ “last supper”^116^ (*Henkersmahlzeit*). Once again, antibiotics featured less as a selector for microbial resistance than as an invisible adulterant. Similar reports continued to appear throughout the German media during 1980s.^117^ Concerns about the risk posed by “infectious resistance” continued to be displaced by a vernacular risk emphasis on residues.

Unsurprisingly, heightened public *Chemie* criticism increased conflicts with West German farmers. Despite often harboring private health concerns, the majority of farmers felt that there was no alternative to chemical-dependent intensification.^118^ This was also true for agricultural antibiotics. While farming magazines stigmatized farmers caught abusing veterinary drugs as “fixers”^119^ and criticized antibiotic use to compensate substandard production methods, they encouraged antibiotic use to optimize management and defeat diseases.^120^

Tensions between West German farmers and the wider public peaked during the early 1980s. On New Year's Eve 1979, the Bavarian Broadcasting Service (*Bayerischer Rundfunk*, BR) caused a uniquely German scandal by broadcasting the *Biermösl Blosn's* “evening prayer of the modern farmer” (*Nachtgebet des modernen Landwirts*). The song was a persiflage of the Bavarian hymn (*Bayernlied*):

God be with you, you land of the BayWa, German fertilizer of phosphate

Over your wide-open fields lies *Chemie* from early morning to late at night

And this is how your beets grow and you feed your sow.

Dear Lord stay in heaven, we have Nitrophoska Blue!^121^

Bavaria's farming community was outraged and demanded a full apology from the BR.^122^ However, three months later, Klaus Peter Schreiner published a further persiflage of a popular children's song in the widely read *Süddeutsche Zeitung*:

In March the farmer starts the tractor

and sprays the field assiduously and tenaciously

no small caterpillar, no small plant survives the poison,

in the forest, the stomach of the small bird rises.

In Summer the farmer empties his sack

fertilizes the fruits of which one eats

he knows how to fertilize by heart

from Bayer, von Hoechst and from BASF.

In Autumn the farmer thanks animal medicine

Because of penicillin, the milk does not turn sour

The Pigs are lean and long as never before

to the honor of animal pharmacology.

In Winter the farmer takes his checkbook

with wife and child he climbs into the Mercedes

He drives into the county town—he is not stupid

and shops in the organic *Reformhaus—*he knows exactly why.^123^

Playing on the popular link between antibiotics, *Chemie*, and *Gift*, the persiflage prompted farming magazines to complain about an “evil ‘poison campaign’ of the public”^124^ and publish lyrical counterattacks. Following further criticism of veterinary drugs on national TV, an agricultural commentator bemoaned the endless stream of depictions of “the farmer as a destructor of the environment and poisoner (*Vergifter*) of food.”^125^

In many ways, the controversies of 1980 reveal the extent to which *Chemie* criticism had become the cornerstone of West German protest against intensive agriculture. For activists, the rhetoric umbrella of *Chemie* enabled the simultaneous criticism of chemically distinct substances like antibiotics, hormones, pesticides, herbicides, and fertilisers. *Chemie's* invisible and poisonous image also enabled powerful analogies to contemporary mass protests against nuclear energy and growing fears about the *Waldsterben*.^126^

However, the power of *Chemie*-focused criticism also meant that West German measures against bacterial resistance proliferation remained half-hearted. The growing international risk episteme surrounding resistance proliferation failed to effectively “stage” bacterial resistance and penetrate West Germans’ vernacular risk discourse. Whereas Sweden pioneered a complete AGP ban because of resistance concerns in 1986, West German authorities continued to limit antibiotic hazards to a “*Gift* in meat problem.”^127^ Because regulating this “*Gift* problem” mainly entailed enforcing withdrawal times, it was both easier and politically more rewarding to tackle than controversial antibiotic restrictions. This did not mean that Germans were unafraid of bacterial resistance proliferation in agricultural settings. Rather, resistance fears paled in comparison to the specter of invisible *Chemie* in milk, food, and bodies.^128^

## Conclusion

In the end, West Germany never effectively addressed agricultural antibiotics’ selection for bacterial resistance. With consumers and farmers embroiled in *Chemie*-conflicts, rising levels of bacterial resistance remained an elephant in the room. Everybody was aware of it but nobody cared enough to priorities it as a risk and regulatory issue.

Real change occurred only after reunification with East Germany where researchers like Wolfgang Witte were already warning about resistance selection on farms.^129^ During the 1990s, the BSE crisis and resistance against reserve antibiotics forced Europeans to reform agricultural antibiotic policies. In 1994, Germany supported the EU's ban of chloramphenicol AGPs. One year later, reports of cross-resistance between avoparcin and the reserve antibiotic vancomycin prompted Germany, Denmark, and the Netherlands to veto a British re-licensing request for avoparcin dairy cow feeds. Germany subsequently followed Denmark's suit in banning avoparcin AGPs in 1996 and supported a 1997 EU ban. With Scandinavian countries pressing for further antibiotic restrictions, the EU decided to phase out virginiamycin, avilamycin, salinomycin-natrium, and flavophospholipol AGPs by July 1999.^130^ The grace period for the four remaining AGPs did not last long. The 2001 confirmation of BSE in German herds resulted in a national crisis and a Green Minister of Agriculture who promptly announced an agricultural turnaround (*Agrarwende*) and supported further antibiotic bans at the European level. Two years later, EU Directive Nr. 1831/2003 led to the phasing out of remaining AGPs by January 2006.

Although the era of antibiotic growth promotion has ended, therapeutic antibiotics remain popular on German farms. Their use is also more widespread than previously thought. Recent data collection reforms have revealed that Germans used 1706 tons of agricultural antibiotics in 2011—the highest amount in Europe.^131^ Although agricultural antibiotic use has since fallen to 1452 tons in 2013, larger amounts of less potent antibiotics have been substituted with smaller amounts of more potent—and therapeutically valuable—fluoroquinolones.^132^

In this situation, the history of antibiotic use has lessons to offer. With bacterial resistance continuing to rise and the pipeline for new antibiotics stalling, regulators will have to reduce antibiotic dependency in both medicine and food production. Despite being invaluable therapeutics, it seems dubious to subsidize the development of new antibiotics if these will merely serve as temporary plugs for a leaking ship. Instead of focusing on technical fixes, regulators will have to redress the cultural root causes of antibiotic overuse in Western medicine and food production. With over half of global antibiotics used in agriculture,^133^ changes in current farming practices are necessary if global antibiotic use is to fall. Moreover, it will not be enough to reduce antibiotic use unilaterally—bacterial resistance knows no borders. Although the 1960s Swann bans and contemporary EU bans had important signal effects, only binding international treaties on reductions of antibiotic use will be able to preserve existing and future antibiotics’ efficacy.

Such a consensus will be difficult to reach. As has been shown for West Germany, there is nothing “natural” about being more concerned about bacterial resistance than about antibiotic residues in food. As highlighted by Ulrich Beck, knowing about a risk is not the same as being sufficiently afraid to regulate it. Instead, the successful cultural “staging” of a risk by experts, activists, and policy makers as well as a risk's successful adoption into consumers’ and producers’ vernacular risk repertoire is decisive for the creation of sustained societal action. In the case of agricultural antibiotics, future regulators will have to do more than merely state the all too familiar “facts” about antibiotic resistance to convince governments to reduce antibiotic use. Instead, they will have to learn and speak in the language of individual risk cultures before being able to successfully “stage risk” and convince consumers, farmers, and officials that substance restrictions are in their long-term self-interest. The nature of this challenge is not only scientific but also cultural.

## Footnotes

1 ‘Die Rache aus dem Stall’, *Zeit* (48/2014).

2 ‘Diese Keime töten’, *ZeitOnline*, 20.11.2014, URL: http://www.zeit.de/wissen/gesundheit/2014-11/multiresistente-keime-mrsa-antibiotika-massentierhaltung-keimkarte (accessed: 20.04.2015); ‘Das bringt uns noch um’, *Zeit* (48/2014), pp. 21–24; Christian Fuchs: ‘Dauernd Stoff vom Arzt’, *ZeitOnline*, 27.11.2014, URL: http://www.zeit.de/2014/49/antibiotika-im-fleisch-tiermedizin/komplettansicht (accessed: 20.04.2015).

3 ‘Landwirte und Tierärzte protestieren vor Zeit-Gebäude’, *Zeitonline*, 28.11.2014, URL: http://www.zeit.de/hamburg/politik-wirtschaft/2014-11/antibiotika-bauerverband-protest (accessed: 20.04.2015); ‘Für uns nicht akzeptabel’, *ZeitOnline*, 17.12.2014, URL: http://www.zeit.de/2014/52/landwirtschaftsminister-christian-schmidt-massentierhaltung-antibiotika-politik (accessed: 20.04.2015); ‘Dart 2020. Antibiotika-Resistenzen Bekämpfen Zum Wohl Von Mensch Und Tier’ (Bundesregierung Deutschland, 2015).

4 Ulrich Beck, *Weltrisikogesellschaft. Auf Der Suche Nach Der Verlorenen Sicherheit* (Frankfurt am Main: Suhrkamp, 2007), pp. 22–36.

5 Ulrich Beck, ‘The Reinvention of Politics: Towards a Theory of Reflexive Modernization’, in Ulrich Beck (ed.), *Reflexive Modernization. Politics, Tradition and Aesthetics in the Modern Social Order* (Cambridge and Oxford: Polity Press, 1995), p. 9.

6 Beck, *Weltrisikogesellschaft. Auf Der Suche Nach Der Verlorenen Sicherheit*, p. 36.

7 Christian Simon, *Ddt. Kulturgeschichte Einer Chemischen Verbindung* (Basel: Christian Merian Verlag, 1999), Natalie Jas, ‘Public Health and Pesticide Regulation in France before and after Silent Spring’, *History and Technology*, 23/4 (2007), Carsten Reinhardt, ‘Boundary Values’, in Viola Balz et al. (eds.), *Precarious Matters/Prekäre Stoffe. The History of Dangerous and Endangered Substances in the 19th and 20th Centuries* (Berlin: Max Plack Institute for the History of Science, 2008 [Preprint]), Nancy Langston, *Toxic Bodies. Hormone Disruptors and the Legacy of Des* (New Haven and London: Yale University Press, 2010), Karin Zachmann, ‘Atoms for Peace and Radiation for Safety—How to Build Trust in Irradiated Foods in Cold War Europe and Beyond’, *History and Technology*, 27/1 (2011), Sarah A. Vogel, *Is It Safe? Bpa and the Struggle to Define the Safety of Chemicals* (Berkeley at al.: University of California Press, 2013).

8 Sheila Jasanoff, *Designs on Nature: Science and Democracy in Europe & the United States* (2 edn.; Princeton and Oxford: Princeton University Press, 2007), p. 9, Alexander Von Schwerin, ‘Prekäre Stoffe: Radiumökonomie, Risikoepisteme Und Die Etablierung Der Radioindikatortechnik in Der Zeit Des Nationalsozialismus’, *NTM. International Journal of History & Ethics of Natural Sciences Technology & Medicine*, 17 (2009), p. 6.

9 Arwen P. Mohun, *Risk. Negotiating Safety in American Society* (Baltimore: Johns Hopkins University Press, 2013), pp. 3–6.

10 Ulrike Thoms, ‘Between Promise and Threat. Antibiotics in Foods in West Germany 1950–1980’, *NTM*, 20 (2012), Heiko Stoff, ‘Vital Regulators of Efficiency: The German Concept of Wirkstoffe, 1900–1950’, in Alexander Von Schwerin, Heiko Stoff, and Bettina Wahrig (eds.), *Biologics, a History of Agents Made from Living Organisms in the Twentieth Century* (London and Brookfield: Picerking and Chatto, 2013), Alexander Von Schwerin, ‘Vom Gift Im Essen Zu Chronischen Umweltgefahren. Lebensmittelzusatzstoffe Und Die Risikopolitische Institutionalisierung Der Toxikogenetik in Der Bundesrepublik, 1955–1964’, *Technikgeschichte*, 81/3 (2014), Heiko Stoff, ‘Zur Kritik Der Chemisierung Und Technisierung Der Umwelt. Risiko-Und Präventionspolitik Von Lebensmittelzusatzstoffen in Den 1950er Jahren’, ibid, Heiko Stoff, ‘Oestrogens and Butter Yellow: Gendered Policies of Contamination in Germany 1930–1970’, in Teresa Ortiz-Gómez and Maria Jesús Santesmases (eds.), *Gender and Well-Being: Gendered Drugs and Medicine. Historical and Socio-Culture Perspectives* (Farnham: Ashgate, 2014), Heiko Stoff, *Gift in Der Nahrung. Zur Genese Der Verbraucherpolitik Mitte Des 20. Jahrhunderts* (Stuttgart: Franz Steiner Verlag, 2015).; the paper also builds on non-German histories of agricultural antibiotic use in Mark R. Finlay, ‘Hogs, Antibiotics, and the Industrial Environments of Postwar Agriculture’, in Philip Scranton and Susan R. Schrepfer (eds.), *Industrializing Organisms. Introducing Evolutionary History* (Hagley Perspectives on Business and Culture; London: Routledge, 2004), Robert Bud, *Penicillin: Triumph and Tragedy* (Oxford: Oxford University Press, 2009), pp. 163–90, Kendra Smith-Howard, ‘Antibiotics and Agricultural Change: Purifying Milk and Protecting Health in the Postwar Era’, *Agricultural History Society*, 84/3 (2010), Thoms, ‘Between Promise and Threat. Antibiotics in Foods in West Germany 1950–1980’.

11 Stoff, *Gift in Der Nahrung. Zur Genese Der Verbraucherpolitik Mitte Des 20. Jahrhunderts*, p. 12.

12 ‘Verbraucherschutz—Behörde hält Chlorhühnchen für Unbedenklich’, *SpiegelOnline*, 10.06.2014, URL: http://www.spiegel.de/gesundheit/diagnose/chlorhuehnchen-ist-laut-bfr-und-efsa-nicht-gesundheitsschaedlich-a-974342.html (accessed: 05.05.2015).

13 Bud, *Penicillin: Triumph and Tragedy*, pp. 168–69, Finlay, ‘Hogs, Antibiotics, and the Industrial Environments of Postwar Agriculture’, pp. 244–54.

14 Alois Seidl, *Deutsche Agrargeschichte* (Frankfurt a. M.: DLG Verlag, 2006), p. 271; 75; 77. Uwe Petersen, ‘Entwicklungen Im Deutschen Futtermittelrecht’, in Bundesforschungsanstalt Für Landwirtschaft (Fal) (ed.), *Meilensteine Für Die Futtermittelsicherheit: Vortragsveranstaltung Im Forum Der Fal Am 16./17. November 2006* (Braunschweig: Petersen, Uwe et al., 2007), p. 3.

15 Cathryn Carson, ‘Bildung Als Konsumgut: Physik in Der Westdeutschen Nachkriegskultur’, in Dieter Hoffmann (ed.), *Physik Im Nachkriegsdeutschland* (Frankfurt: Harri Deutsch, 2003).

16 Bayerisches Hauptstaatsarchiv (Bavarian Main State Archive)[In the following HSTA] Ministerium für Ernährung Landwirtschaft und Forsten (MELF)-365 B22-1159/5641 UA. Betriebswirtschaft und Agrarpolitik an Ministerialrat Dr. Nichterlein (22.12.1952); ‘Der Fortschritt in der Landwirtschaft’, supplement of the *Bayerisches Landwirtschaftliches Wochenblatt* (BLW) (2/1950); ‘Amerikanischer Austausch-Jungbauer. Ben Coleman traf in Bayern ein’, *BLW* (31/1950), p. 62; ‘Farmerleben in Neu-England (Amerika)’, *BLW* (35/1950), p. 667.

17 ‘Tierische Erzeugnisse bestimmen den Erlös’, *BLW* (18/1955), p. 797; HJ Teuteberg, ‘Die Entwicklung des Fleischverbrauchs in Deutschland’, *GESIS HISTAT: historische Statistik*, ZA8303, (1972 [2008]), URL: http://www.gesis.org/histat/de/search (accessed: 21.04.2015); Thoms, ‘Between Promise and Threat. Antibiotics in Foods in West Germany 1950–1980’, p. 189.

18 Finlay, ‘Hogs, Antibiotics, and the Industrial Environments of Postwar Agriculture’, pp. 239–44; HSTA Ministerium des Innern (MInn) 87547, Bd.I, 5568 a5, Office of the US High Commission for Germany to Bayerisches Staatsministerium des Innern (06.03.1950); 5568 Lederle Laboratories Division to Wilhelm Pschorr (06.10.1949); HSTA MELF -366, 6004/c5 Prof. Dr. Sommer an Ministerialdirektor Dürrwaechter (17.01.1958), pp. 7–8; Thoms, ‘Between Promise and Threat. Antibiotics in Foods in West Germany 1950–1980’, p. 198.

19 G. Fiegenbaum, ‘Mutterlose Ferkelaufzucht—eine einfache und preiswerte Methode’, *LWWL* (10/1964), p. 26; D. Lohmann, ‘Vorbeugung ist besser als Heilen’, *LWWL* (18/1966), pp. 88–89; ‘Lomacin—Commercial: Risiko-minderung’, *LWWL* (40/1969), p. 2.

20 HSTA MInn 87762 Anlage zu III 6b – 5594/2–3/66 Deutscher Bundestag – 5. Wahlperiode; Drucksache V/907, p. 16.

21 HSTA MInn 91348/II Anlage zu IAI 23-2-2/470 Vorschlag der Kommission für eine Richtlinie des Rats über Zusatzstoffe in der Tierernährung (Bundesrat; Direktor Drucksache 410/67, Anlage I, pp. 8–9.

22 ‘Alarm im Schweinestall’, *Zeit* (13/1968), pp. 40–41.

23 ‘Nichts dem Zufall überlassen’, *BLW* (02/1970) (special supplement), p. 23.

24 ‘Leckerbissen für Viren’, *Der Spiegel* (13/1954), p. 36; ‘Chemie des Lebens’, *Zeit* (28/1958), p. 4; ‘Gefährlicher Papageienschmuggel über Belgien und Holland’, *Frankfurter Allgemeine Zeitung* (FAZ), 06.09.1956, p. 8.

25 ‘Schock nach der Spritze’, *Spiegel* (30/1954), pp. 30–31; ‘Wissenschaft kämpft für das Leben’, *Zeit* (41/1958), p. 5.

26 ‘Antibiotika im Kotelett’, *FAZ*, 08.08.1956, p. 13.

27 Thoms, ‘Between Promise and Threat. Antibiotics in Foods in West Germany 1950–1980’, p. 184.

28 Stoff, ‘Zur Kritik Der Chemisierung Und Technisierung Der Umwelt. Risiko-Und Präventionspolitik Von Lebensmittelzusatzstoffen in Den 1950er Jahren’, pp. 233–39, Stoff, ‘Vital Regulators of Efficiency: The German Concept of Wirkstoffe, 1900–1950’, Robert N. Proctor, *The Nazi War on Cancer* (Princeton and Oxford: Princeton University Press, 2000), pp. 17–19; 71; 122–41, Gunter Vogt, ‘The Origins of Organic Farming’, in W. Lockeretz (ed.), *Organic Farming. An International History* (Wallingford and Cambridge (M.A.): CABI, 2011).Gunter Vogt, ‘The Origins of Organic Farming’, in W. Lockeretz (ed.), *Organic Farming. An International History* (Wallingford and Cambridge (M.A.): CABI, 2011).

29 Ulrike Thoms, ‘Vegetarianism, Meat, and Life Reform in Early Twentieth-Century Germany and Their Fate in the ‘Third Reich”, in David Cantor and Christian Bonah (eds.), *Meat, Medicine and Health in the Twentieth Century* (Studies for the Society for the Social History of Medicine; London: Pickering & Chatto, 2010), p. 157, Frank Uekötter, *The Green and the Brown: A History of Conservation in Nazi Germany* (Cambridge: Cambridge University Press, 2006).

30 Stoff, ‘Zur Kritik Der Chemisierung Und Technisierung Der Umwelt. Risiko-Und Präventionspolitik Von Lebensmittelzusatzstoffen in Den 1950er Jahren’, Alexander Von Schwerin, ‘Vom Gift Im Essen Zu Chronischen Umweltgefahren. Lebensmittelzusatzstoffe Und Die Risikopolitische Institutionalisierung Der Toxikogenetik in Der Bundesrepublik, 1955–1964’, ibid, pp. 254–56; 61–66, Stoff, ‘Oestrogens and Butter Yellow: Gendered Policies of Contamination in Germany 1930–1970’, pp. 30–32. Martin Wiebel, ‘Meine Lebensmittel für Europa’, *FAZ*, 07.08.1958, p. 2.

31 HSTA MELF-1149, III/5424 BMELF an die Mitglieder des Verbraucherausschusses für Ernährungsfragen, Anlage—Deutsche Gesellschaft für Ernährung. Leitsätze für eine vollwertige Ernährung Februar 1956 (05.07.1956), p. 5.

32 Curt Lenzner, *Gift in Der Nahrung. Eine Warnung, Zugleich Ein Aufruf Zur Rückkehr Zu Unverfälschter Nahrung* (2nd [1931] edn.; Leipzig: Mehnert & Pleyner 1933), Thoms, ‘Between Promise and Threat. Antibiotics in Foods in West Germany 1950–1980’, p. 193.

33 HSTA MELF-1150 III/7281 BMELF and Mitglieder des Verbraucherausschusses für Ernährungsfragen, Anlage: Niederschrift über die 18. Sitzung des Verbraucherausschusses für Ernährungsfragen vom 07.11.1956 in Bonn (03.01.1957), p. 7.

34 Thoms, ‘Between Promise and Threat. Antibiotics in Foods in West Germany 1950–1980’, p. 191, Stoff, *Gift in Der Nahrung. Zur Genese Der Verbraucherpolitik Mitte Des 20. Jahrhunderts*, pp. 149–75.

35 ‘Antibiotische Futterzusätze’, *FAZ*, 21.10.1959, p. 7; on Kollath see: Jörg Melzer, *Vollwerternährung, Diätetik, Naturheilkunde, Nationalsozialismus, Sozialer Anspruch* (Stuttgart: Franz Steiner Verlag, 2003), pp. 214–19; 49; 71, Stoff, ‘Vital Regulators of Efficiency: The German Concept of Wirkstoffe, 1900–1950’, pp. 101–04, Stoff, ‘Zur Kritik Der Chemisierung Und Technisierung Der Umwelt. Risiko-Und Präventionspolitik Von Lebensmittelzusatzstoffen in Den 1950er Jahren’, pp. 247–48, Gunther Viereck, ‘"Laßt Das Natürliche So Natürlich Wie Möglich" – Der Hygieniker Und Ernährungswissenschaftler Werner Kollath (1892–1970)’, in Gisela Boeck and Hans-Uwe Lammel (eds.), *Die Universität Rostock in Den Jahren 1933–1945* (Rostock: Universität Rostock, 2012).

36 Stoff, ‘Oestrogens and Butter Yellow: Gendered Policies of Contamination in Germany 1930–1970’, pp. 32–34, Langston, *Toxic Bodies. Hormone Disruptors and the Legacy of Des*, pp. 77–82.

37 ‘Gift in der Nahrung’, *Spiegel* (45/1958), p. 37; on Kulturkritik and its use by contemporary experts see: Stoff, *Gift in Der Nahrung. Zur Genese Der Verbraucherpolitik Mitte Des 20. Jahrhunderts*, p. 12.

38 ‘Gift in der Nahrung’, *Spiegel* (45/1958), p. 45.

39 HSTA MELF-1150, III-751 BMELF an die Mitglieder des Verbraucherausschusses für Ernährungsfragen und die ständigen Gäste—Niederschrift der 21. Sitzung des Verbraucherausschusses für Ernährungsfragen am 20.01.1958 (31.03.1958), p. 10.

40 Ibid, p. 13.

41 Ibid, p. 14.

42 ‘Gift In Der Nahrung’, *Spiegel* (45/1958), p. 45; ‘Eßwaren ohne Tarnung’, *FAZ*, 29.08.1959, p. BuZ6.

43 HSTA MELF-1150, 5000/86-2507 Berufsorganisation der Hausfrauen e.v. an Staatsminister Hundhammer (02.06.1958), p. 3.

44 ‘Gift In Der Nahrung’, *Spiegel* (45/1958), p. 46; S. Walter Souci, ‘Zur Problematik Der Lebensmittelkonservierung Unter Aspekten Internationaler Zusammenarbeit’, *Angewandte Chemie*, 71/5 (1959), pp. 184–85.

45 Scott H. Podolsky et al., ‘History Teaches Us That Confronting Antibiotic Resistance Requires Stronger Global Collective Action’, *Journal of Law, Medicine & Ethics*, 43/2 (Special Supplement) (2015); on the adaptationist controversy see Angela N. H. Creager, ‘Adaptation or Selection? Old Issues and New Stakes in the Postwar Debates over Bacterial Drug Resistance’, *Studies in History and Philosophy of Biology and Biomedical Sciences*, 38 (2007), p. 159.

46 Thoms, ‘Between Promise and Threat. Antibiotics in Foods in West Germany 1950–1980’, pp. 184–85; 90.

47 Ibid, p. 189. West German attendees of the 1955 International Conference on Antibiotics in Agriculture were Munich University's Johannes Brüggemann and Enno Freerksen, director of the tuberculosis research institute Borstel (Bad Oldesloe), ‘Proceedings First International Conference on the Use of Antibiotics in Agriculture’, *First International Conference On Antibiotics In Agriculture* (National Academy of Sciences – National Research Council, 1955), pp. 9–10.

48 ‘Fassung des Arzneimittelgesetzes vom 24.08.1976’, Bundesgesetzblatt [BGBL] I Nr. 109, 31.08.1976, pp. 2445, 2448, 2483; for a more extensive study of German pharmaceutical law see Arthur Daemmrich, *Pharmacopolitics. Drug Regulation in the United States and Germany* (Chapel Hill: University of North Carolina Press, 2011).

49 Petersen, ‘Entwicklungen Im Deutschen Futtermittelrecht’, pp. 3–4.

50 ‘Alarm im Schweinestall’, *Zeit* (13/1968), p. 41.

51 Ibid, pp. 40–41.

52 H. Williams Smith and W. E. Crabb, ‘The Effect of the Continuous Administration of Diets Containing Low Levels of Tetracyclines on the Incidence of Drug-Resistant Bacterium Coli in the Faeces of Pigs and Chickens: The Sensitivity of the Bact. Coli to Other Chemotherapeutic Agents’, *The Veterinary Record*, 69 (1959).

53 E. S. Anderson, ‘Origin of Transferable Drug-Resistance Factors in the Enterobacteriaceae’, *British Medical Journal*, 2/5473 (1965), E. S. Anderson and Naomi Datta, ‘Resistance to Pencillins and Its Transfer in Enterobacteriaceae’, *The Lancet*, 285/7382 (1965), E. S. Anderson and M. J. Lewis, ‘Drug Resistance and Its Transfer in Salmonella Typhimurium’, *Nature*, 206/4984 (1965).

54 Bud, *Penicillin: Triumph and Tragedy*, pp. 174–80, Claas Kirchhelle, *Pyrrhic Progress. Antibiotics and Western Food Production (1949–2013)* (Oxford [Dissertation], 2015).

55 Hans Knothe, ‘Langfristige Nutritive Anwendung in Der Tierernährung Im Hinblick Auf Die Menschliche Gesundheit’, *Münchener Medizinische Wochenschrift*, 105/4 (1963), pp. 175–81.

56 ‘Die Antibiotika-Mast und die Gesundheit’, *FAZ*, 02.07.1963, p. 9.

57 Hans-Joachim Metzlaff, ‘Werden die Tiere zu rasch schlachtreif gemacht’, *FAZ*, 23.02.1963, p. 7.

58 Ibid.

59 Ruth Harrison, *Tiermaschinen. Die Neuen Landwirtschaftlichen Fabrikbetriebe* (München: Biederstein, 1965).

60 ‘Angst vor der biologischen Bombe’, *Spiegel* (21/1966), p. 65; also see: HSTA Minn 87782 Anlage zu III – 5594/2-11/65 Deutscher Bundestag, Niederschrift der Sitzung anläßlich der Fragestunde, Bonn, 29.11.1965, pp. 81–82.

61 ‘Bakterien. Fühler des Schreckens’, *Spiegel* (31/1967), p. 101.

62 Ibid.

63 ‘Alarm im Schweinestall’, *Zeit* (13/1968), pp. 40–41.

64 ‘So vergiften wir unsere Umwelt’, *Zeit* (28/1969), p. 45; also see the reformed Hans Knothe, ‘Die Epidemiologie Der Salmonellosen’, *Zentralblatt für Bakteriologie, Parasitenkunde, Infektionskrankheiten und Hygiene – Erste Abteilung*, 205/1-3 (1967).

65 ‘Beim Pflanzenschutz sich selbst schützen’, *BLW* (12/1965), p. 68; ‘Chemischer Pflanzenschutz’ und Wild’, *LWWL* (20/1966), pp. 7–9; ‘Das ist auch im Hausgarten wichtig: Vorsicht mit Pflanzenschutzmitteln’, *BLW* (19/1965), p. 38.

66 ‘Nach der Penicillin-Behandlung: Fünf Tage lang keine Milch liefern’, *LWWL* (43/1965), p. 16; Teschner, ‘Rentable Hühnerhaltung nur bei gesunder Herde’, *LWWL* (32/1963), p. 2232.

67 Kirchhelle, *Pyrrhic Progress. Antibiotics and Western Food Production (1949–2013)*.

68 ‘Alarm im Schweinestall’, *Zeit* (13/1968), p. 42.

69 Helmut Kewitz, ‘Antibiotika in Der Tierernährung’, *Zentralblatt für Veterinärmedizin*, 17 (1970 (Series B)), p. 116; the exact relation between the Kewitz and Swann Committees is the subject of ongoing research on the part of the author.

70 Kewitz, ‘Antibiotika in der Tierernährung’, pp. 117–18.

71 Ibid, p. 118.

72 HSTA MInn 91348/I IA-1 25-1/338 Bundesrat an den Bundeskanzler und den Herren Präsidenten des Bundesrates (26.06.1967); in November 1963 a harmonisation of antibiotic rules had explicitly been rejected by Council Directive (64/54/EEC); on the enactment of the Kewitz recommendations see Löbsack, ‘Veruntreute Arzneimittel’, *Zeit* (13/1971).

73 HSTA MInn 91348/II 24-7/131Bundesrat-Gesundheitsausschuß No. 9 7126; Niederschrift über 26. Sitzung am 28.08.1967; Punkt 3 EWG Vorlage 338/67; Directive 70/524/EEC (23.11.1970).

74 Kewitz, ‘Antibiotika in Der Tierernährung’, p. 119.; J. I. R. Castanon, ‘History of the Use of Antibiotic as Growth Promoters in European Poultry Feeds’, *Poultry Science*, 86/11 (2007), pp. 2446–69.

75 ‘Drogen im Futter, Gift auf dem Tisch’, *Spiegel* (26/1971).

76 ‘Auf Wachstum getrimmt’, *Spiegel* (26/1971), p. 54.

77 ‘Wenn sie nicht fressen, spritze ich sie selbst’, *Spiegel* (26/1971), p. 52.

78 Ibid, p. 46.

79 Ibid.

80 Ibid., pp. 49 & 62.

81 Theo Löbsack, ‘Veruntreute Arzneimittel’, *Zeit* (13/1971).

82 ‘Die Migros Polizei’, *FAZ*, 02.11.1971, p. 13; ‘Was ein Filetstück heutzutage alles enthalten kann’, *FAZ*, 15.03.1972, p. 10; ‘Gefährliche Medikamente im Kochtopf?’, *FAZ*, 11.01.1971, p. 7.

83 HSTA MInn (vorläufig) 5580 BA2 Antibiotika und Östrogene, Verfügung A; Karton Nr. 10.2/10.3, Aktenband I Arzneimittel und Östrogene; Beiakt zu Akten-Nummer 5580, Abdruck aus der Süddeutschen Zeitung Nr. 43, 22.02.1972, p. 9; Abdruck aus Münchener Merkur – Münchener Stadtzeitung Nr. 43, 22.02.1972, p. 11.

84 HSTA MInn (vorläufig) 5580 BA2 Antibiotika und Östrogene, Verfügung A; Karton Nr. 10.2/10.3, Aktenband I Arzneimittel und Östrogene; Beiakt zu Akten-Nummer 5580, II 13-5325 h-M-1/72 Landeshauptstadt München, Kommunalreferent an die Regierung von Oberbayern (29.02.1972); Entwurf III 6- 5580/13 an das Bayerische Staatsministerium für ELF (24.03.1972); IE6-5580/20 Regierung von Oberfranken an das Bayerische Staatsministerium des Innern (07.06.1973).

85 HSTA MInn (vorläufig) 5580 BA2 Antibiotika und Östrogene, Verfügung A; Karton Nr. 10.2/10.3, Aktenband I Arzneimittel und Östrogene; Beiakt zu Akten-Nummer 5580, Anlage 4: III 6-5580/16, Entwurf an den BMJFG (25.04.1972), p. 2.

86 HSTA MInn (vorläufig) 5580 BA 2 Hemmstoffe – Östrogene, Thyreostatika in Lebensmitteln, Verfügung A; 10.1, III6 – 5580/4-1 Bayerische Staatliche Veterinäruntersuchungsanstalt Nürnberg an das Bayerische Staatsministerium des Innern (08.03.1972).

87 HSTA MInn (vorläufig) 5580 BA2 Antibiotika und Östrogene, Verfügung A; Karton Nr. 10.2/10.3, Aktenband I Arzneimittel und Östrogene; Beiakt zu Akten-Nummer 5580, IE5-4480/E1 7.77i Der Minister für ELF des Landes Nordrhein-Westfalen an den BMJFG (28.06.1972), p. 1.

88 HSTA MInn (vorläufig) 5580 BA 2 Hemmstoffe – Östrogene, Thyreostatika in Lebensmitteln, Verfügung A; 10.1, III 6 – 5683/146 Bayerische Landesanstalt für Tierseuchenbekämpfung an das Bayerische Staatsministerium des Innern (02.11.1971), p. 2; Memorandum Abteilung IE – Antibiotika im Fleisch [undated], pp. 4–5.

89 ‘Fleischbeschaulich’, *Spiegel* (33/1972), p. 58; HSTA MInn (vorläufig) 5580 BA2 Antibiotika und Östrogene, Verfügung A; Karton Nr. 10.2/10.3, Aktenband I Arzneimittel und Östrogene; Beiakt zu Akten-Nummer 5580, IE6 Bu11.9 Information des BJFG (31.08.1972), p. 2; on Käte Strobel and residue detections in foreign meat see: Stoff, *Gift in Der Nahrung. Zur Genese Der Verbraucherpolitik Mitte Des 20. Jahrhunderts*, pp. 75–76 & 78. HSTA MInn (vorläufig) 5580 BA 2 Hemmstoffe – Östrogene, Thyreostatika in Lebensmitteln, Verfügung A; 10.1, III 6 – 5683/63 Entwurf an die Regierungen (24.05.1971); III 6 – 5683/132 Entwurf an den BMJFG (30.09.1971).

90 HSTA MInn (vorläufig) 5580 BA 2 Hemmstoffe – Östrogene, Thyreostatika in Lebensmitteln, Verfügung A; 10.1, Memorandum Abteilung IE – Antibiotika im Fleisch [undated], pp. 1 & 9.

91 Ibid, p. 10.

92 ‘Verordnung zur Änderung der Ausführungsbestimmungen A über die Untersuchung und gesundheitspolizeiliche Behandlung der Schlachttiere und des Fleisches bei Schlachtungen im Inland vom 18.12.1973’, *Bundesgesetzblatt* (BGBL) I Nr. 1/74, p. 18; on clashes between researchers and industry and government experts see: HSTA MInn (vorläufig) 5580 BA 2 Hemmstoffe – Östrogene, Thyreostatika in Lebensmitteln, Verfügung A; 10.1, Gottfried Wolff an Hans Eisenmann (26.09.1972); Landwirtschaftliche Untersuchungs- und Forschungsanstalt Kiel an Schiedermaier (28.06.1972); Institut für Ernährungsphysiologie in der Tierärztlichen Fakultät (LMU) an Schiedermaier (28.06.1972); Gottfried Wolff an G. Beck (27.09.1972).

93 ‘Biologischer Hemmstofftest’, *Bayerisches Landesamt für Gesundheit und Lebensmittelsicherheit*, URL: http://www.lgl.bayern.de/lebensmittel/hygiene/fleischhygiene/hemmstofftest/ (accessed: 05.05.2015).

94 ‘Hemmstoff-Test am Ende’, *BLW* (26/1975), p. 9; on testing in the US see ‘Regulation of Food Additives and Medicated Animal Feeds’, *Subcommittee of the Committee on Government Operations* (House of Representatives Washington DC: US Government Printing Office 1971), p. 183.

95 ‘Der Hemmstofftest ist ein Lotteriespiel’, *LWWL* (36/1973), p. 44.

96 HSTA MInn (vorläufig) 5580 BA 2 Hemmstoffe – Östrogene, Thyreostatika in Lebensmitteln, Verfügung A; 10.1, LUFA Kiel an Schiedermaier (28.06.1972), p. 2.

97 ‘Deutsche Nahrungsmittel werden zunehmend gesünder’, *LWWL* (41/1974), p. 14.

98 The British National Archives (TNA) MAF 461/67 (Steering Group on Food Surveillance Sub-Group on Antibiotic Residues in Food, 1st Meeting, 20 April, 1976), pp. 5 and 7.

99 TNA MAF 461/68 (EEC, Summary Report of Meeting with Representatives of Community Institutions or Of Member Governments, ‘Working Party‚ Veterinary Legislation’ Sub-Group‚ Residues’ (25–26.05.1978), ‘Draft Directives on Undesirable Residues in Fresh Meat’, 30 May, 1978), p. 2.

100 Ibid.

101 Theo Löbsack, ‘Veruntreute Arzneimittel. Antibiotika im Futter’, *Zeit* (13/1971); the earliest reference to *Autobahntierarzt* found after a cursory search is ‘Neuer Schlag gegen Tierärzte’, *BLW* (50/1975), p. 12.

102 HSTA MInn (vorläufig) 5581 Hausapotheken der Tierärzte, Karton 12 (1968–1974), Bayerischer Landtag 8. Wahlperiode Drucksache 8/338 (12.03.1975); Münsterer, ‘Tierärztliche Betreuung neu geregelt’, *BLW* (35/1975), p. 8.

103 HSTA MInn (vorläufig) Hausapotheken der Tierärzte (Karton 12), 1976–1987 Band V, IE 6 – 5581/4 Regierung der Oberpfalz an das Bayerische Staatsministerium des Innern (22.08.1977), p. 2; also see: Hausapotheken der Tierärzte (Karton 12), 1976–1987 Band V, Anlage zu 5581/7 H. Kahn an das Veterinäramt Mayen-Koblenz (03.03.1977).

104 HSTA MInn (vorläufig) Hausapotheken der Tierärzte (Karton 12), 1976–1987 Band V, IE 6 – 5581/4 Regierung der Oberpfalz an das Bayerische Staatsministerium des Innern (22.08.1977), p. 3.

105 HSTA MInn (vorläufig) Hausapotheken der Tierärzte (Karton 12), 1976–1987 Band V, IE6-5581-4/84 Bayerisches Staatsministerium des Innern – Protokoll der Besprechung am 28.03.1984 zum Thema ‘Tierärztliche Hausapotheke in der Gemeinschaftspraxis der Herren Dr. Schmidt, Dr. Lang und Wörner (10.07.1984), p. 3; also see: Hausapotheken der Tierärzte (Karton 12), 1976–1987 Band V, Anlage zu 5581/7 Dr. H. Kahn an das Veterinäramt Mayen-Koblenz (03.03.1977).

106 HSTA MELF-173e 7132/1837 Anhang: Bundeskriminalamt an den Bundesminister des Innern (02.05.1981), pp. 1–2.

107 *BGBL* I (03.09.1968), p. 990; HSTA MELF-173b (vorläufig), 7132/1524 Anlage: Bayerische Landesanstalt für Tierzucht an den Ministerialdirigent Minderle, Anlage –Schärferes Futtermittelrecht (03.01.1974); MELF (vorläufig) -173a, 7132/1470 BMELF an die Herren Minister und Senatoren für ELF der Länder – Anlage Schreiben des BMELF an den Bundesminister für Justiz (29.01.1974);MELF (vorläufig) -173a, 7132/1519 Entwurf Abteilung M an das Bundes MELF (13.02.1975); MELF-173 b (vorläufig) 7132/1620 Bayerische Landesanstalt für Bodenkultur und Pflanzenbau an das Bayerische Landesamt für Ernährungswirtschaft z.Hd. Herrn Jany –Amtliche Futtermittelkontrolle – Hier Fütterungsantibiotika (15.09.1976).

108 HSTA MELF (vorläufig) -173a, 7132/1472 Bayerische Landesanstalt für Bodenkultur und Pflanzenbau an das Bayerische Staatsministerium für ELF – Bericht zur mikrobiologischen Antibiotika-Analytik 1973 (01.02.1974), p. 1.

109 HSTA MELF-173d (vorläufig) 7132/1766 Der Hessische Minister für Landesentwicklung, Umwelt, Landwirtschaft und Forsten an Herrn Landwirtschaftsattaché Holger Bunk Dybdahl, Staatskonsulat Kg. Dansk Ambassade (30.10.1970), p. 2; MELF-173b (vorläufig), 7132/1587 Bayerisches Landesamt für Ernährungswirtschaft, Bericht über die amtliche Futtermittelkontrolle im Kalenderjahr 1975 (24.02.1976), p. 3.

110 HSTA MELF-173c (vorläufig), 7132/1677 BMELF an die Minister und Senatoren für ELF (10.11.1977), p. 5; West Germany had already supported EEC AGP licensing for Hoechst's Flavomycin; HSTA MELF-173c (vorläufig) 7132/1659 BMELF an Niedersächsischen Minister für ELF – Anhang 1 Auszug aus dem Bericht über die 29. Sitzung des Sachverständigenausschusses 06–07.09.1976 (03.06.1977).

111 Thoms, ‘Between Promise and Threat. Antibiotics in Foods in West Germany 1950–1980’, pp. 200–01; on organic agriculture see: Frank Uekötter, *Die Wahrheit Ist Auf Dem Feld. Eine Wissensgeschichte Der Deutschen Landwirtschaft* (Göttingen: Vandenhoeck & Ruprecht, 2010), pp. 413–17, Warren J. Belasco, *Appetite for Change. How the Counterculture Took on the Food Industry* (2nd edn.; Ithaca and New York: Cornell University Press 2007), pp. 68–69, Kirchhelle, *Pyrrhic Progress. Antibiotics and Western Food Production (1949–2013)*.

112 ‘Horn und Ernährung’, *Spiegel* (46/1971), p. 67; cf. also: Sybil Gräfin Schönfeldt, ‘Antibiotische ‘saure Nieren”, *Zeit* (39/1973), p. 61; ‘Zeitlese – Glücksschwein’, *Zeit* (9/1989), p. 87; Horst Dohm, ‘Zehn Quadratmeter Wiese für jedes Huhn’, *FAZ*, 03.04.1980, p. 14; Peter Brügge, ‘Das vergiftete Paradies’, *Spiegel* (44/1978), p. 100.

113 Katalyse-Umweltgruppe Köln, *Chemie in Lebensmitteln* (Köln: Zweitausendeins, 1982), pp. 11–13.

114 Frank Uekötter and Claas Kirchhelle, ‘Wie Seveso Nach Deutschland Kam. Umweltskandale Und Ökologische Debatten Von 1976 Bis 1986.’, *Archiv für Sozialgeschichte*, 52 (2012), pp. 333–34.

115 ‘Vergiften uns die Bauern?’, *Spiegel* (44/1978).

116 Peter Brügge, ‘Das vergiftete Paradies’, *Spiegel* (44/1978), p. 99.

117 ‘Hauptsache es hilft und fällt nicht auf’, *Spiegel* (44/1980), pp. 112–119; ‘Mengen von illegalen Tierarzneimitteln’, *FAZ*, 28.10.1981, p. 9; ‘Tierarzneimittel für Millionen illegal verkauft’, *FAZ*, 15.09.1984, p. 7; Reiner Klingholz, ‘Schweinerei im Kälberstall’, *Zeit* (34/1988), p. 1.

118 ‘Dobler greift Verbraucher Arbeitsgemeinschaft an’, *LWWL* (04/1974), p. 12; ‘Landwirte fürchten die Folgen der verschärften Umweltschutzbestimmungen’, *LWWL* (03/1974), p. 10; Seidl, *Deutsche Agrargeschichte*, pp. 293–94; on private health fears see Ilse Witte, ‘Wohin führt uns der technische Fortschritt?’, *LWWL* (45/1973), p. 9); Anna Brunner, ‘Gift ist keine Lösung’, *LWWL* (31/1970), p. 5; Reinold Dohmann, ‘Vergiften wir uns selbst?’, *LWWL* (42/1973), p. 6; ‘Gift ist keine Lösung’, *BLW* (31/1970), p. 5; Eisenmann, ‘Nur keine ‘Chemie’ ins Bier’, *BLW* (48/1975), p. 12.

119 ‘Östrogenmast wurde ein teurer Spaß’, *LWWL* (2/1973), p. 16; ‘Neuer Schlag gegen Tierärzte’, *BLW* (50/1975), p. 12; ‘So kann kranken Mastschweinen häufig geholfen werden’, *LWWL* (05/1976), p. 31.

120 ‘Nichts dem Zufall überlassen’, *BLW* (02/1970) (special supplement), p. 23; ‘Antibiotika gegen Milchfieber bei Schweinen’, *BLW* (40/1970), p. 6.

121 W.N., ‘Chemie, Chemie’, *BLW* (02/1980), p. 11.

122 ‘Protest gegen BayWa Hymne’, *BLW* (07/1980), p. 8; the BR subsequently apparently imposed a broadcasting ban on the *Biermösl Blosn*; the band later caused another scandal by accidently being engaged to play at the annual strong beer festival (*Starkbierfest*) of the conservative Bavarian Christian Social Union, ‘Ein Trio gegen die Tümelei’, *Zeit* (22/1987), p. 51.

123 Quoted according to ‘Liebe Leser’, *BLW* (14/1980), p. 2.

124 ‘Die Killer Story’, *BLW* (27/1980), p. 11; ‘Verunsicherte Verbraucher – diskriminierte Landwirte’, *BLW* (16/1980), p. 4.

125 ‘Giftmischer’, *BLW* (22/1980), p. 11.

126 Links between Western fears of ‘invisible’ contamination by chemicals and radioactivity have been pointed out by numerous authors, Ralph H. Lutts, ‘Chemical Fallout: Rachel Carson's Silent Spring, Radioactive Fallout, and the Environmental Movement’, *Environmental Review*, 9/3 (1985), Kendra Smith-Howard, *Pure and Modern Milk. An Environmental History since 1900* (Oxford: Oxford University Press, 2013), Schwerin, ‘Vom Gift Im Essen Zu Chronischen Umweltgefahren. Lebensmittelzusatzstoffe Und Die Risikopolitische Institutionalisierung Der Toxikogenetik in Der Bundesrepublik, 1955–1964’.

127 HSTA MInn (vorläufig) 5580 BA 2 Hemmstoffe – Östrogene, Thyreostatika in Lebensmitteln, Verfügung A; 10.1, Gottfried Wolff to Ltd. Ministerialrat Dr. Hans Schiedermaier (03.10.1972); a 1989 DFG ad hoc group on resistance had no effect on agricultural practice ‘Senatskommission Zur Prüfung Von Rückständen in Lebensmitteln’, *Deutsche Forschungsgemeinschaft Jahresbericht* (Bonn, 1988).

128 Early examples of agricultural resistance-focussed criticism are Rainer Flöhl, ‘Die ‘Problem-Infektion’ und die Antibiotika’, *FAZ*, 14.11.1973, p. 33; ‘Resistente Salmonellen in der Nahrungskette’, *FAZ*, 03.10.1984, p. 33.

129 Ruth Hummel, H Tschäpe, and Wolfgang Witte, ‘Spread of Plasmid Mediated Nourseothricin Resistance Due to Antibiotic Use in Animal Husbandry’, *Journal of Basic Microbiology*, 26 (1986).

130 Dettenkofer, M.; Ackermann, M.; Eikenberg, M.; Merkel, H., *Auswirkungen Des Einsatzes Von Antibiotika Und Substanzen Mit Antibiotischer Wirkung in Der Landwirtschaft Und Im Lebensmittelsektor. Ein Literatur-Review* (Ernährungs Wende Produkte, Materialienband Nr. 4; Freiburg: Institut für Umweltmedizin und Krankenhaushygiene am Universitätsklinikum Freiburg, 2004), p. 24 & 27; Helmut Bündner, ‘Von glücklichen Bauern und gedopten Milchkühen’, *FAZ* (21.07.1995), p. 15; ‘Verbot des Antibiotikums ‘Avoparcin’ in Futtermitteln’, *European Commission Press Release* ip/97/71 (30.01.1997); Bud, *Penicillin: Triumph and Tragedy*, pp. 192–203, 06, 08.

131 ‘Europa-Vergleich: Deutschland führt bei Antibiotika-Einsatz in der Tiermast’, *SpiegelOnline*, 26.09.2012, URL: http://www.spiegel.de/wirtschaft/service/deutschland-ist-spitzenreiter-bei-antibiotika-einsatz-in-tiermast-a-858092.html (accessed: 27.04.2015); ‘Germap 2012’ (Rheinbach Bundesamt für Verbraucherschutz und Lebensmittelsicherheit, Paul-Ehrlich-Gesellschaft für Chemotherapie e.V., Infektiologie Freiburg, 2014 (April)), pp. 5–6.

132 ‘Dart 2020. Antibiotika-Resistenzen Bekämpfen Zum Wohl Von Mensch Und Tier’, pp. 9–10.

133 ‘Non-therapeutic Use of Antibiotics in Animal Agriculture, Corresponding Resistance Rates, and What Can be Done About It’, *Center for Global Development*, 19.06.2009, URL: http://www.cgdev.org/article/non-therapeutic-use-antibiotics-animal-agriculture-corresponding-resistance-rates-and-what (accessed: 05.10.2015).

